# Knowledge, attitudes and perceptions of stroke: a cross-sectional survey in rural and urban Uganda

**DOI:** 10.1186/s13104-015-1820-6

**Published:** 2015-12-26

**Authors:** Mark Kaddumukasa, James Kayima, Martin N. Kaddumukasa, Edward Ddumba, Levi Mugenyi, Svetlana Pundik, Anthony J. Furlan, Martha Sajatovic, Elly Katabira

**Affiliations:** Department of Medicine, School of Medicine, Makerere University College of Health Sciences, P.O. Box 7072, Kampala, Uganda; Department of Medicine, St Raphael of St Francis Nsambya Hospital, Nkozi University, P.O. Box 7146, Kampala, Uganda; Infectious Diseases Research Collaboration, Mulago Hill Road, MUJHU3 Building, P.O. Box 7475, Kampala, Uganda; University Hospitals Case Medical Center, Neurological Institute Case Western Reserve University, 11100 Euclid Avenue, Cleveland, OH 44106 USA; Neurological and Behavioral Outcomes Center, University Hospitals Case Medical Center, 11100 Euclid Avenue, Cleveland, OH 44106 USA

**Keywords:** Stroke, Knowledge, Risk factors, Warning factors

## Abstract

**Background:**

Information regarding the increasing burden of non-communicable diseases such as stroke is largely unknown among the vulnerable communities. This analysis, which is part of a larger U.S. National Institute of Heath-funded Medical Education Partnership Initiative neurological disorder survey, assessed community knowledge and attitudes on stroke and stroke risk factors.

**Methods:**

A population cross-sectional survey was conducted in urban and rural Mukono, district, central Uganda. Through the systematic sampling method, data were gathered from 377 adult participants who were interviewed about selected aspects of stroke knowledge, attitudes and perception using a pretested structured questionnaire.

**Results:**

A total of 377 participants were enrolled (47 % urban). The leading risk factors identified by the participants were stress (36.6 %) and hypertension (28.9 %) respectively. None of the study participants identified cigarette smoking as a stroke risk factor. Seventy six percent of the participants did not recognize stroke as a disease of the brain.

**Conclusion:**

Stroke knowledge is poor in both rural and urban Uganda. Tailored public health approaches that improve stroke awareness, knowledge and self management approaches are urgently needed to develop effective preventive measures and community response to stroke.

**Electronic supplementary material:**

The online version of this article (doi:10.1186/s13104-015-1820-6) contains supplementary material, which is available to authorized users.

## Background

### Background/rationale

Stroke is one of the leading causes of mortality and morbidity worldwide with developing countries accounting for 85 % of global deaths from stroke [1–4]. The lack of correct medical information and poor control of stroke risk factors contributes significantly to the rising incidence of stroke amongst Africans [5, 6]. Community attitudes and knowledge influence stroke prevention including risk factor identification and management as well as community and individual response to stroke symptoms when they occur. The success of primary preventive measures and timely medical attention immediately following a stroke is influenced by the public’s knowledge and perception of stroke and its risk factors [5, 7]. Few studies have been conducted in sub-Saharan Africa, with over half of them in Nigeria. However the awareness of stroke, and its risk factors and symptoms is low in community studies conducted in African studies [8, 9]. While in Nigerian studies among university staff, students and health workers, the awareness of stroke risk factors was high [5, 6].

In Uganda, the public’s understanding and beliefs of stroke, its warning signs and associated risk factors have not been well studied. Nakibuuka and colleagues [10] recently conducted a survey on stroke risk factors and warning signs in community-dwelling individuals. This study [10] found that nearly 3/4 of study participants were unable to identify stroke risk factors and warning signs and did not recognize stroke as a brain disorder [10]. Understanding the knowledge gaps and perceptions of stroke are critical to inform and lead to the development of appropriate targeted health promotion campaigns to prevent stroke among high risk populations in our setting. This study objectives were therefore to assess the knowledge and perceptions of stroke among urban and rural populations in Mukono district, central Uganda. This assessment, was part of larger study on neurological disorder knowledge and attitudes, provided context on how the community perceived neurological disorders more broadly.

## Methods

### Study design and population

This study was part of a larger U.S. National Institute of Heath-funded Medical Education Partnership Initiative (MEPI) neurological disorder survey, where we assessed community knowledge and attitudes on stroke and stroke risk factors among some of the study participants.

This was a cross-sectional study conducted within an ongoing population survey of 3000 participants on prevalence and incidence of neurological diseases in Mukono district. Face to face interviews were conducted between August and November 2014. Multistage stratified sampling technique was used as described below [11]. At the subcounty level, urban Mukono Town Council (TC) was randomly selected and Nakisunga sub–county as rural were randomly selected out of 13 sub-counties. Mapping of the selected urban and rural areas was based on the Uganda Population and Housing census where 11,373 and 9570 households were identified in Mukono TC and Nakisunga respectively [12]. The sampling frame was all households in these areas. Systematic sampling was used to select households in each village to total 2000 in the urban area and 2200 in the rural area that would participate in the large population survey. Out of the 1500 participants in the urban area and 1500 in the rural area, systematic sampling technique was used to select every tenth household for this interview. If the selected household objected participation, then the next household would be considered. A total of 377 participants participated in this study with 177 and 200 from the urban and rural areas, respectively. They were interviewed on selected aspects of stroke knowledge, attitudes and perception. The Inclusion criteria included; usual resident who is present in the sampled household on the night before the survey, aged 18 years or older (adult) and willingness to provide informed consent. We excluded those who were physically unable to undergo interview. One adult, randomly selected from each household was approached and consented, participated in the study. Only one participant was randomly selected from each household using simple random method. We employed randomly selected cards by the potential participants written on “Yes” and “No” for study participation. If similar cards were drawn then the process would be repeated until one participant was selected. The selected households were visited by the research team. The randomly selected participant was informed about the research and the intended use of the information obtained. A request for a written informed consent was then sought. To address potential sources of bias, a standardised questionnaire was used with a wide range of responses for the study participants which were read to the participants. The study interviewers received trained on the study protocols for data collection in order to minimize inter-observer variability during data gathering and entering data.

### Sample size determination

The sample size calculation for stroke knowledge and attitudes was based on the prevalence of hypertension which us an important risk factor for stroke. The sample size was calculated using formula: { $n\; = \;\frac{{Z_{\alpha }^{2} (pq)}}{{d^{2} }}$ } where p = prevalence of hypertension, q = complement of the prevalence, margin of error is error = d, α = significance level. Setting significance at 0.05 and error margin at 5 %, adjusted sample requirement for an assumed 10 % level of non-response (nr = 10 %) = N*. Based on a previous study in Mukono [13] where hypertension prevalence was 27 % and N* = 336, we recruited 370 participants.

### Questionnaire and measurements

We used a modified standardized questionnaire that assessed knowledge and attitudes towards stroke already used in the sub-Sahara African settings [5, 10, 14] (see Additional file 1). Participant’s knowledge of stroke warning signs was categorized based on the numbers of stroke warning signs [15, 16]. Individuals with good knowledge could identify 5–10 stroke warning signs, fair knowledge 2–4 signs, and poor knowledge one stroke warning sign. A similar categorization was used for participant’s knowledge of stroke risk factors.

### Ethical considerations

Ethical approval for the study was obtained from Makerere University College of Health Sciences’ School of Medicine review board and ethics committee Ref number 2013-145 and UNCST Ref Number. HS 1551. Written informed consent was obtained before enrolling the participants into the study.

### Data analysis

Descriptive statistics of mean, frequency, and percentages were used to summarise data on socio-demographic variables and stroke knowledge and perceptions. Chi square or Fisher’s exact tests were used as appropriate to assess associations between stroke knowledge and perception and demographic variables and self-reported risk factors. Logistic regression was used to determine predictors of knowing the organ affected by stroke, good level of knowledge of stroke warning signs and risk factors. All tests of hypothesis were two tailed with a level of significance at 0.05. All statistical analysis was performed using STATA software version 12 (Stata Corporation, College Station, TX, USA).

## Results

**Fig. 1:**
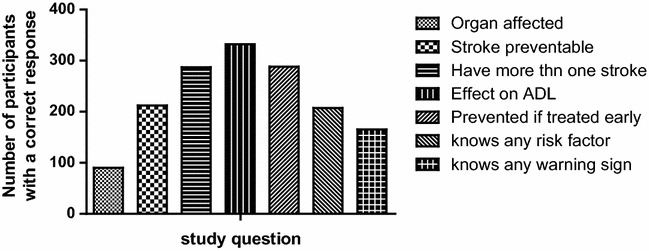
Shows the number of study respondents with the correct answers to the study questions. Less than one-third of the study respondents correctly knew the brain as the site affceted by stroke

A total of 377 study participants with the urban settings contributing 177 (47 %) adults were enrolled into this study. Sixty eight percent (260/377) of the study participants were women with a median age (IQR) of 34 (26–48) years. The age of the study participants ranged from 18–85 years. Other demographics are shown in Table 1. The number of study participants who correctly reponsed to the study questions is shown in Fig. 1.

**Table 1:** Demographic characteristics of the study participants (N = 377)

Characteristic	
Median age in years (IQR)	34 (26–48)
Age range in years (min, max)	18–85
Age categories in years, n (%)	
<25	74 (19.7)
25–34	123 (32.8)
35–44	66 (17.6)
>44	112 (29.9)
Gender, n (%)	
Male	117 (32.0)

### Organ affected by stroke and risk factors

**Fig. 2:**
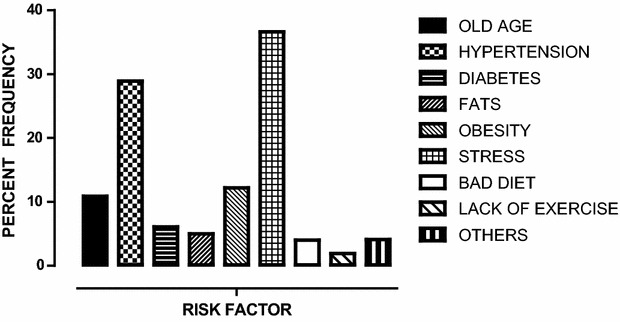
Shows the frequency of reported known risk factors among the study participants. Stress was the leading known risk factor reported followed by hypertension by the study respondents

Only 24 % (90/377) of participants correctly identified stroke as a disorder of the brain. The majority (59.4 %) did not have any idea of the organ affected by stroke. Nearly 16 % (59/377) thought that the heart is the site of stroke (Table 2). Two hundred and seven participants (73.4 %) knew at least one risk factor for stroke (Fig. 2). The most common risk factor identified by 138 (36.6 %) participants was stress (43 % urban; 31 % rural; P = 0.016) followed by hypertension 109 (28.9 %; 36 % urban; 23 % rural; P = 0.004). No participants identified cigarette smoking as a stroke risk factor.

**Table 2:** Knowledge of stroke disease (N = 377)

Study question	N (%)
Organ of the body affected by stroke	
Brain	90 (23.9)
Heart	59 (15.7)
Lungs	4 (1.1)
Liver	0 (0)
Don’t know	224 (59.4)
Is stroke preventable	
Yes	212 (56.2)
No	83 (22.0)
Don’t know	82 (21.8)
Can one have stroke more than once	
Yes	287 (76.1)
No	31 (8.2)
Don’t know	59 (15.7)
Does stroke have an effect on daily activities like driving a car, dressing, having job etc	
Yes	332 (88.1)
No	14 (3.7)
Don’t know	31 (8.2)
Can stroke be prevented if treated early	
Yes	288 (76.4)
No	21 (5.6)
Don’t know	68 (18.0)
Knows any risk factors for stroke	
Yes	207 (54.9)
No	10 (2.7)
Don’t know	160 (42.4)
Knows any warning factors for stroke	
Yes	165 (43.8)
No	18 (4.8)
Don’t know	194 (51.5)

Recognizing stroke as a disorder of the brain was not, however, associated with knowledge of stroke warning signs, P = 0.746. Using a simple logistic regression, identifying stroke as a brain disorder was associated with knowing that stroke is preventable, possibly recurrent, that early treatment can prevent stroke with p values of 0.002, 0.01 and 0.005 respectively. Knowing that stroke can be prevented if treated early and if a person can have more than one stroke were not associated with knowledge regrading stroke risk factors. with p values; 0.280 and 0.138, respectively.

### Warning symptoms of stroke

**Fig. 3:**
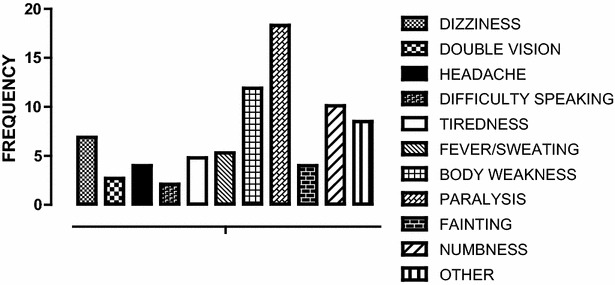
Shows the knowledge for the warning signs of stroke among the study participants. Paralysis and body weakness were the common warning signs reported by the study participants

Two hundred and fifteen participants (57 %) knew no stroke warning signs or symptoms (Fig. 3). Eighteen percent (69/377) reported paralysis as the leading warning sign of stroke, followed by body weakness 12 %, (45/377); numbness 10 % (38/377) respectively. Of the 162 (43 %) that knew stroke warning signs, 84 (52 %) knew 1 warning sign, 76 (47 %) knew 2–4 warning signs, and 2 (1 %) knew five or more warning signs (Table 3). At multivariable logistic regression, we analysed for the factors associated with knowledge of stroke warning signs. Residence of the study participants with P value of 0.04 with adjusted OR (95 % CI) of 1.72 (1.03–2.90) and knowing that stroke is preventable, P value = 0.07; adjusted OR (95 % CI), 0.08 (0.04–0.15) remained statistically significant. While knowing any risk factor for stroke was not associated with the knowledge of stroke warning signs, P value = 0.133.

**Table 3:** Factors associated with knowledge for warning signs of stroke

	Knows one or more warning signs for stroke, N (%)	Don’t know any warning sign for stroke, N (%)	P
Residence			
Rural	76 (46.9)	124 (57.7)	0.038
Urban	86 (53.1)	91 (42.3)	
Age in years			
<25	30 (18.6)	44 (20.6)	0.331
25–34	46 (28.6)	77 (36.0)	
35–44	32 (19.9)	34 (15.9)	
>44	53 (32.9)	59 (27.6)	
Sex			
Male	48 (30.8)	69 (32.9)	0.672
Female	108 (69.2)	141 (67.1)	
Stroke preventable			
Yes	83 (51.2)	129 (60.0)	0.000
No	61 (37.7)	22 (10.2)	
Don’t know	18 (11.1)	64 (29.8)	
Person can have stroke more than once			
Yes	135 (83.3)	152 (70.7)	0.000
No	18 (11.1)	13 (6.1)	
Don’t know	9 (5.6)	50 (23.3)	
Stroke has effect on daily activities			
Yes	155 (95.7)	177 (82.3)	0.000
No	4 (2.5)	10 (4.7)	
Don’t know	3 (1.9)	28 (13.0)	
Stroke can be prevented if treated early			
Yes	130 (82.3)	158 (73.5)	0.068
No	11 (6.8)	10 (4.7)	
Don’t know	21 (13.0)	47 (21.9)	
Knows risk factors for stroke			
3 or more	31 (19.1)	10 (4.7)	0.000
1 or 2	115 (80.0)	63 (29.3)	
Don’t know	16 (9.9)	142 (66.1)	
Knows Organ affected by stroke			
Yes	40 (24.7)	50 (23.3)	0.746
No	122 (75.3)	165 (76.7)	

### Stroke prevention, recurrence, and effect on activities of daily living

More than half of particiapants 56 % (212/377) reported that stroke is preventable. Nearly two-thirds (76 %, 287/377) believed that stroke can be recurrent. Eighty eight percent of participants (332/377) reported that stroke can have an effect on daily activities like driving a car, dressing, and having a job.

## Discussion

This survey of stroke knowledge and attitudes conducted in urban and rural Uganda found that many individuals have extremely limited knowledge of risk factors for stroke, are not aware that stroke is a brain disease, and are not familiar with many of the common early warning signs and symptoms of stroke. Individuals residing in rural setting were most likely to be poorly informed about stroke. The findings of this report are very much in line with another recent report also conducted in the Ugandan setting (10). Majority of the study participants were young which is in keeping with the Ugandan population structure with a median age of 15.6 years [12].

With the rising burden of non-communicable diseases in sub–Saharan Africa, there is a need to develop strategies to prevent them. Lack of knowledge or incorrect knowledge of stroke risk factors and lack of awareness of the warning signs contributes to the rising incidence of stroke amongst Africans [5, 14]. The dissemination of accurate information regarding stroke risk factors and warning signs is important to prevent stroke morbidity and fatality within vulnerable communities. Increasing stroke knowledge among communities is known to result in a shorter time of presentation to the emergency department following stroke onset [17].

None of the study participants identified smoking as an important risk factor for stroke yet smoking has been reported as an important risk factor. Second hand smoke has also been reported to increase the odds of stroke by six fold [18, 19]. The Ugandan national prevalence of smoking has been rising to currently 16.6 % [20]. Providing correct information regarding smoking addressing its relationship with stroke and other chronic diseases would be key within our communities.

Stress was reported as the leading cause of stroke more in the urban populations than rural. Stress is associated with cardiovascular disease and metabolic syndromes all which increase the risk of strokes [21, 22]. Identifying personal stressors and addressing them is critical in reducing this risk.

Our study reports that only 47.2 % individuals knew at least one of the known risk factors of stroke. This is comparable to other studies performed else where [5, 10, 23]. In these other reports less knowledgable individuals tended to be from poorer communities and had less education. In our analysis, rural residents, where education and income levels are generally expected to be lower, had less stroke awareness. Only 28.9 % of the study participants cited hypertension as a stroke risk factor. Hypertension, is one of the most important risk factors for stroke [24]. This is slightly lower than earlier studies which have reported higher proportions of 32–51 % in the general public [16, 25–29]. The identification of hypertension, in these earlier studies may be attributed to the differences in health care systems, awareness among these populations. Nevertheless, considering the intimate relationship between hypertension and stroke, future stroke awareness campaigns and health education need to emphasize the significance of hypertension. While the lack of stroke knowledge in our sample is daunting, it also provides an opportunity to spur action, and can inform stroke awareness campaigns in Uganda [30]. Tailored stroke public health campaigns are essential for Ugandan rural adults to enhance their knowledge regarding stroke symptomatology and risk factors. These tailored campaigns should include packages of self management approaches and key messages as only increasing knowledge regarding stroke alone has been found to be ineffective if there is no reinforcement [31–33]. This will help them in the future to reduce stroke risk, identify stroke should it occur, and mobilize individuals and the community to respond appropriate to stroke early warning signs. Finally, identifying high-risk sub-groups (for example, individuals with severe and poorly controlled hypertension) and instituting appropriate measures might help mitigate the rising threats of stroke in Uganda and sub Saharan Africa.

### Limitations

Our study is limited by the fact that it was cross-sectional, used close-ended questions, and was confined to a fairly limited geographic area. This might have limited the responses regarding knowledge and attitudes to stroke omiting some of the respondents’ responses. However, the survey questions were worded such that the answer choices covered a wide range of response possibilities and findings seem to confirm similar data obtained in another recent study on stroke awareness in Uganda.

## Conclusion

Future stroke awareness strategies should emphasize that stroke is a brain disease, that it is preventable, and help individuals to understand and manage stroke risk factors. Considering the importance of prompt treatment in improving patient outcomes, we must continue to advise individuals and communities with regard to appropriate action during emergencies. Finally, the most important implication of the present study is the need to focus on primary and secondary prevention, and crucially, target low-income high risk subjects.

## Additional file

10.1186/s13104-015-1820-6 The study questionnaire.

## Abbreviations

TC: town council
MEPI: medical education partnership initiative
