# Supplementary material for: Knowledge, attitudes and perceptions of stroke: a cross-sectional survey in rural and urban Uganda
Source: BMC Res Notes. 2015 Dec 26;8:819. doi: 10.1186/s13104-015-1820-6 (PMC4691295; doi:10.1186/s13104-015-1820-6)
Supplement: Supplementary file 1 — 10.1186/s13104-015-1820-6 The study questionnaire. [file 13104_2015_1820_MOESM1_ESM.docx]

Participant ID Initials

|  |  |  |  | - |  |  |
| --- | --- | --- | --- | --- | --- | --- |

| Section 1: Demographics |
| --- |

1.1 Interviewer name & code no.…………………………………

1.2 Date of interview:

1.3 Residence codes:

1.4 Was written Informed Consent obtained? 🞏 No 🞏 Yes

*If no, please do not proceed.*

1.6 Gender: 🞏 Male 🞏 Female

1.7 Date of birth:

If year of birth not known ask or estimate age (years) |__|__| AGE

1.8 Marital status: 🞏 Married

🞏 Single- never married

🞏 Divorced

🞏 Separated

🞏 Widowed

1.9 Religion: 🞏 Catholic

🞏 Protestant

🞏 Muslim

🞏 Pentecostal

🞏 Traditional

🞏 Other………..

1.10 Highest level of education attained: 🞏 None

🞏 Primary (P1-7)

🞏 Secondary (S1-6)

🞏 Tertiary (University)

| Section 2: Knowledge about stroke |
| --- |

2.1 What organ of the body is affected by stroke: 🞏 Brain 🞏 Heart

🞏 Kidney 🞏 Liver

🞏 Lungs 🞏 Don’t know 🞏 Other…………………

2.2 Is stroke preventable? :🞏 Yes 🞏 No

2.3 Can a person have stroke more than once? :🞏 Yes 🞏 No

2.4 Does stroke have an effect on daily activities like driving a car, dressing, use of the toilet and having a job? : 🞏 Yes 🞏 No

| What do you believe causes a stroke? - |
| --- |

🞏 Demons 🞏 hypertension 🞏 don’t know

🞏 Witch craft 🞏 cigarette smoking 🞏 Bad diet

🞏 God’s will 🞏 Fatty foods 🞏 alcohol

🞏 Atherosclerosis 🞏 high cholesterol 🞏 Stress

🞏 Angry ancestral spirits 🞏 Obesity

🞏 Oral contraceptives 🞏 lack of exercise

🞏 Inheritance

🞏 Others (please specify)…………………………………………………………

| **What do you believe are risk factors for stroke?** |
| --- |

3.1 Do you know any risk factors for stroke? 🞏 Yes 🞏 No

If Yes, what are the risk factors for stroke that you know of? Please tick all that applies

🞏 Old age 🞏 hypertension

🞏 Diabetes 🞏 cigarette smoking

🞏 Heart disease 🞏 alcohol

🞏 Atherosclerosis 🞏 high cholesterol

🞏 Obesity 🞏 genetics (hereditary)

🞏 Stress 🞏 lack of exercise

🞏 Poor hygiene 🞏 headache or migraine

🞏 Cancer 🞏 oral contraceptives

🞏 Bad diet 🞏 tremors

🞏 Others

| **Knowledge of stroke warning signs** |
| --- |

3.2 Do you know any warning signs of stroke? 🞏 Yes 🞏 No

3.3 If Yes, what are the warning signs of stroke that you know of? Please tick all that applies

🞏 Dizziness 🞏 blurred or double vision or loss of vision

🞏 Headache 🞏 sudden difficulty in speaking or understanding or reading

🞏 Tiredness 🞏 fever/sweating

🞏 Shortness of breath 🞏 Chest pain or chest tightness

🞏 Nausea/vomiting 🞏 weakness of any part of the body

🞏 Weakness of one side of the body 🞏 paralysis of any part of the body

🞏 Paralysis of one side of the body 🞏fainting black out collapse

🞏 Numbness tingling sensation or dead sensation of any body part

🞏 Numbness tingling sensation or dead sensation of one side of the body

🞏Others (please specify…………...

| What would be your planned response to an event of stroke? |
| --- |

🞏 Call general practitioner or family doctor

🞏 Ask family members or relatives to help

🞏 Go to chemist for advice or medication

🞏 Self medication

🞏 Ask friend or neighbours for help

🞏 Go to hospital

🞏 Visit community health centre

🞏 Visit alternative health care providers (herbal med, traditional healers),

🞏 Seek spiritual healing (prayer)

🞏 Combination of hospital and tradition

🞏 Combination of hospital and faith

🞏 Invite a Physiotherapist

🞏 Others (please specify)

| **Sources of information about stroke** |
| --- |

What are your sources of information about stroke? Please tick all that applies

🞏 Health care providers 🞏 Friends and relatives

🞏 Radio 🞏 TV 🞏 News papers

🞏 Electronic media 🞏 Others (please specify)…………
